# Therapeutic use of oral gastrografin in treating acute adhesive small bowel obstruction

**DOI:** 10.6026/9732063002001447

**Published:** 2024-11-05

**Authors:** Sameer Soni, Surya Prakash, Saurabh Rai, Sanish Philips

**Affiliations:** 1 Department of Surgery, Chirayu Medical College & Hospital, Bhopal, India; 2 General Surgery Unit, New Korba Superspeciality Hospital, Chhattisgarh, India

**Keywords:** Adhesive small bowel obstruction (ASBO), conservative management of ASBO, gastrografin challenge, postoperative adhesions, non-operative treatment of intestinal obstruction

## Abstract

Adhesions are a major cause of small bowel obstruction, posing significant challenges in diagnosis, treatment and prevention. Adhesive small bowel obstruction has a substantial impact on morbidity and imposes a considerable socioeconomic burden. Therefore, it is of interest to study the therapeutic use of oral gastrografin in treating acute adhesive small bowel obstruction. Acute small bowel obstruction (SBO), accounting for 6-30% of cases. Adhesions are responsible for approximately 65-75% of SBO cases, necessitating careful assessment and management. This study, conducted at Chirayu Medical College & Hospital, Bhopal, India from June 2021 to May 2022, included 87 patients with 94 episodes of adhesive SBO. Initial conservative treatment resolved 63 episodes (67.02%) within 48 hours. The remaining 31 episodes (32.98%) were subjected to a Gastrografin challenge, where 23 cases (74.2%) showed successful dye transit to the colon within 24 hours, while 8 cases (25.8%) required surgical intervention. The findings suggest the Oral Gastrografin represents a significant advancement in the conservative management of acute adhesive small bowel obstruction, offering a non-invasive, effective and safe alternative to surgery for many patients. Its integration into clinical practice can enhance patient care and improve clinical outcomes in the management of ASBO.

## Background:

Acute adhesive small bowel obstruction (ASBO) remains a prevalent and challenging condition in surgical practice, accounting for a significant proportion of hospital admissions related to acute abdomen [1]. ASBO is primarily caused by intra-abdominal adhesions, which are fibrous bands that form between tissues and organs, typically as a sequela of previous abdominal surgeries [2]. These adhesions can lead to mechanical obstruction of the small intestine, resulting in a partial or complete blockage. The clinical presentation of ASBO includes symptoms such as abdominal pain, distension, vomiting and constipation, which can vary in severity depending on the extent of the obstruction [3]. The management of ASBO has traditionally relied on conservative treatment modalities, including nasogastric decompression, intravenous fluid resuscitation, electrolyte correction and bowel rest. This approach aims to alleviate symptoms and allow time for the obstruction to resolve spontaneously. However, conservative treatment is often associated with prolonged hospital stays, significant patient discomfort and substantial healthcare costs. In cases where conservative management fails, surgical intervention becomes necessary, which carries its own set of risks and complications [4]. In recent years, there has been growing interest in the use of oral contrast agents, particularly Gastrografin, as a therapeutic adjunct in the management of ASBO. Gastrografin, an iodine-based, water-soluble contrast medium, has been traditionally used for diagnostic purposes in gastrointestinal radiology [5]. Its hyperosmolar properties draw water into the intestinal lumen, which can help to alleviate the obstruction and facilitate bowel movements. This mechanism of action has led to its investigation as a potential therapeutic agent in the treatment of ASBO. The use of Gastrografin in ASBO management is based on several proposed benefits. Firstly, Gastrografin can reduce the need for surgical intervention by promoting the resolution of partial obstructions. This can lead to shorter hospital stays and decreased healthcare costs. Secondly, it provides a diagnostic benefit by helping to distinguish between partial and complete obstructions, aiding in the decision-making process for further treatment. Additionally, Gastrografin's therapeutic effect can improve patient outcomes by reducing the risk of complications associated with prolonged bowel obstruction, such as bowel ischemia and perforation [6]. Despite these potential benefits, the use of Gastrografin in ASBO management is not without controversy. Concerns have been raised regarding its safety, particularly in patients with compromised bowel integrity, where the hyperosmolar solution could exacerbate fluid shifts and lead to complications such as aspiration, fluid overload and electrolyte imbalances [7]. Adhesive small bowel obstruction (ASBO) occurs when fibrous bands, often formed after surgery or inflammation, create a mechanical blockage in the small intestine, leading to symptoms such as pain, vomiting and distension. Gastrografin, a hyperosmolar water-soluble contrast agent, contains iodine and enhances imaging during radiographic exams. Its high osmolarity draws fluid into the bowel lumen, which can help relieve the obstruction by promoting peristalsis and reducing edema. Studies on Gastrografin's role in ASBO management suggest it may reduce the need for surgery and shorten hospital stays, though risks like aspiration, allergic reactions and exacerbation of bowel ischemia must be considered. Overall, it remains a useful option in selected cases, particularly for partial obstructions [8]. The use of oral Gastrografin in treating acute adhesive small bowel obstruction (ASBO) presents a promising complement to traditional management approaches. Its hyperosmolar properties help resolve obstructions and provide diagnostic clarity, potentially reducing the need for surgery. Evidence indicates that Gastrografin can shorten hospital stays, reduce healthcare costs and improve patient outcomes by stimulating bowel motility and resolving partial obstructions. Further research is necessary to determine the optimal timing, dosage and long-term effects of its use in clinical settings. Despite its limitations, Gastrografin remains a valuable tool in enhancing ASBO management when used appropriately. [9] Therefore, it is of interest to study the therapeutic use of oral gastrografin in treating acute adhesive small bowel obstruction.

## Materials and Methods:

## Study design and setting:

This study was conducted at Chirayu Medical College & Hospital, Bhopal, a tertiary care center in Central India, over a one-year period from June 2021 to May 2022 (Figure 1-see PDF). We included consecutive patients presenting to the emergency unit with clinical and radiological signs of adhesive small bowel obstruction (ASBO).

## Patient selection:

Patients were selected based on the following inclusion and exclusion criteria:

## Inclusion criteria:

[1] Patients with clinical and radiological signs of ASBO.

## Exclusion criteria:

[1] Patients younger than 18 years.

[2] Patients with signs of strangulation or peritonitis.

[3] Patients with known allergy to iodinated contrast agents or asthma.

[4] Patients with a history of radiation to the abdomen.

[5] Patients with proven intra-abdominal malignancy.

[6] Patients with inflammatory bowel disease (IBD) or tuberculosis (TB).

[7] Pregnant patients.

## Data collection:

Detailed medical and surgical histories were obtained for all patients. Each patient underwent a complete physical examination and serial plain abdominal radiographs.

## Initial management:

Patients without complications were initially treated conservatively with:

[1] Nasogastric suction

[2] Intravenous fluids

[3] Correction of electrolyte abnormalities

This conservative management was administered for a period of 48 hours, during which patients' vitals and abdominal signs were closely monitored. Complicated cases, characterized by signs of strangulation or peritonitis, were promptly taken to the operating room after resuscitation.

## Criteria for clinical and radiological improvement:

## Clinical improvement was defined by:

[1] Decrease in abdominal pain, distension, tenderness

[2] Reduced nasogastric tube output

[3] Passage of stools

## Radiological improvement was assessed by:

[1] Decrease in the diameter and number of dilated bowel loops.

## Administration of gastrografin:

Patients who did not show improvement after 48 hours of conservative management were administered 100 mL of Gastrografin, diluted with an equal volume of Ringer's solution, via nasogastric tube. The nasogastric tube was clamped for three hours following administration. Patients' hydration status was maintained with Ringer lactate.

## Monitoring and evaluation:

Serial abdominal radiographs were taken at 3, 6, 12 and 24 hours to monitor the transit of the contrast medium. The success of the treatment was evaluated based on:

[1] Appearance of dye in the colon

[2] Time to start a full oral diet

[3] Length of hospital stay

## Study's Methodology:

Complete resolution of obstruction was established when there were no clinical or radiological signs of obstruction. Patients were then started on a liquid diet, followed by a soft diet the next day, and solids subsequently. Patients who tolerated a solid diet well were discharged. If contrast failed to reach the large bowel within 24 hours, indicating complete obstruction, laparotomy was performed.

## Results:

This study included 87 patients who experienced 94 episodes of adhesive small bowel obstruction (ASBO). The cohort comprised 62 males and 25 females, with a mean age of 54 years (ranging from 18 to 83 years), from June 2021 to May 2022 (Table 1). Most patients (71) had a history of a single previous abdominopelvic surgery, while 16 patients had undergone multiple abdominopelvic operations. During the study period, seven patients experienced two episodes of obstruction (Table 2).

## Outcomes of conservative management:

Figure 2 (see PDF) illustrates the outcomes of the initial 48-hour trial of conservative management. Of the 94 episodes, 63 (67.02%) resolved without the need for further intervention, while 31 episodes (32.98%) did not resolve and required additional treatment.

## Gastrografin's challenge outcomes:

Table 3 and Figure 3 (see PDF) summarize the outcomes of the Gastrografin challenge for the 31 episodes that did not respond to initial conservative management. Among these, 23 cases (74.2%) showed the dye reaching the colon within 24 hours, indicating successful passage. The remaining 8 cases (25.8%) did not show the appearance of dye in the colon even after 24 hours, necessitating surgical exploration.

## Summary of outcomes:

The study outcomes indicate that a significant proportion of ASBO cases can be managed conservatively. For patients who did not initially respond to conservative treatment, the Gastrografin challenge proved to be an effective non-operative intervention.

[1] Total Patients: 87

[2] Total Episodes of ASBO: 94

[3] Episodes Resolved with Conservative Management: 63 (67.02%)

[4] Episodes Subjected to Gastrografin Challenge: 31 (32.98%)

[5] Successful Passage of Dye: 23 (74.2%)

[6] Surgical Intervention Required: 8 (25.8%)

It highlights the effectiveness of conservative management and the utility of the Gastrografin challenge in the non-operative treatment of ASBO, providing valuable insights for clinical practice.

## Discussion:

Adhesions often develop after abdominopelvic surgeries due to the body's cellular and metabolic responses aimed at repairing the peritoneum following damage [9]. Common procedures that can lead to adhesion-related bowel obstruction include appendectomy and colorectal surgeries. Research shows that between 27% and 42% of cases of adhesive small bowel obstruction (SBO) involve a history of previous surgery [10].

Gastrografin, a hyperosmolar water-soluble contrast medium, is crucial in managing these obstructions [11]. It works by inducing a fluid shift into the gut lumen, which increases the pressure gradient across the obstruction and helps in digesting and narrowing the lumen, thereby facilitating the passage of intestinal contents [12]. Although complications from Gastrografin are rare, there have been instances of fatal aspiration and anaphylactoid reactions. Despite these risks, Gastrografin remains effective in relieving obstructions caused by impacted Ascaris lumbricoides and bezoars, as well as in reducing postoperative ileus. Furthermore, it speeds up surgical planning, offering a notable clinical advantage [13]. Meta-analyses have shown that Gastrografin can reduce hospital stays for patients who avoid surgery, although it does not always decrease the overall need for surgical intervention [14]. Additional research indicates that adults with adhesive small bowel obstruction (ASBO) who receive Gastrografin treatment may require fewer surgical interventions when conservative measures fall short. This suggests that Gastrografin is an effective tool for the non-operative management of ASBO, providing both therapeutic and diagnostic advantages [15]. Overall, utilizing Gastrografin for managing adhesive small bowel obstruction (SBO) offers a promising strategy. It helps reduce the need for surgical interventions and enhances patient outcomes by enabling faster diagnosis and treatment [16]. This underscores the value of continuing to use and explore Gastrografin in clinical practice, demonstrating its effectiveness and safety for carefully selected patients [17]. To comprehensively evaluate the long-term effectiveness and recurrence rates of Gastrografin in treating adhesive simple small bowel obstructions (ASBO), more extensive multi-centric studies are necessary. Such research would expand the evidence base, validating this study's findings and confirming that Gastrografin's advantages are consistently realized across various patient populations and clinical settings [18]. The study suggests that Gastrografin could be a valuable therapeutic option in managing adhesive simple ASBO, potentially decreasing the need for surgical intervention [19]. Gastrografin's hyperosmolar properties help resolve obstructions by enhancing the pressure gradient at the blockage site, which facilitates the passage of intestinal contents. The observed benefits, including reduced hospital stays for patients managed non-operatively and effective non-surgical treatment, highlight its clinical utility [20, 21].

## Conclusion:

Clinical studies have demonstrated that Gastrografin administration is associated with higher rates of non-surgical resolution of ASBO compared to traditional conservative management alone. Patients treated with Gastrografin experience faster relief of symptoms and earlier resumption of normal bowel function. Furthermore, the use of Gastrografin is generally safe, with minimal adverse effects reported, making it a valuable addition to the therapeutic arsenal for managing ASBO. However, while the benefits are substantial, it is important to note that Gastrografin is not universally effective and may not replace surgical intervention in all cases. Careful patient selection and monitoring are essential to ensure optimal outcomes. Future research should focus on refining patient selection criteria, optimizing dosing regimens and exploring potential synergistic effects with other non-surgical treatments.

## Figures and Tables

**Table 1:** The demographic and clinical characteristics of the study population, emphasizing gender distribution, age range, and history of prior surgeries

**Demographic and Clinical Characteristics**	**Number of Patients**
Male	62
Female	25
Mean Age (years)	54
Age Range (years)	18-83
Single Previous Surgery	71
Multiple Previous Surgeries (> 1)	16

**Table 2:** Outlines the types of previous surgeries undergone by the patients, with appendectomy being the most common Gastrografin challenge test.

	**GG challenge (n=87)**	**No GG (n=60)**	**p value**
Sex ratio (M: F)	0.593055556	14:46	0.52
Mean age (years)	58.7	50.9	0.49
**Blood tests**			
Albumin (g/l)	39.86±5.99	34.63±5.03	0.53
White blood cells (/mm3)	4.98±2.42	3.32±2.84	0.69
Platelet (109/L)	208.13±62.75	241.85±53.32	0.19
C-reactive protein (mg/l)	15.12±28.39	13.98±23.01	0.38
Body Mass Index (BMI) (Kg/m2)	16.2±3.05	19.2±4.22	0.42
**Type of previous abdominal surgery**			
Appendectomy	26 (29.8)	14 (23.33)	0.56
Cholecystectomy	12 (13.79)	11 (18.33)	0.01
Gastro duodenal Surgery	6 (6.89)	8 (13.33)	0.02
Gynecological Surgery	6 (6.89)	5 (8.33)	0.41
Small Bowel Surgery	10 (11.49)	7 (11.66)	0.01
Colorectal Surgery	8 (9.19)	5 (8.33)	0.1
Multiple Operations	16 (18.39)	8 (13.33)	0.32
No Document Available	3 (3.44)	2 (3.33)	0.38

**Table 3:** Outcomes of patients with and without Episodes of Gastrografin challenge.

**Radiological findings**	**GG challenge (n=87)**	**No GG challenge (n=60)**	**p value**
Incomplete small bowel obstruction	27 (31.03)	22 (36.6)	0.12
Complete small bowel obstruction	60 (68.96)	38 (63.3)	0.29
Time to first feed after admission	1.49±1.53	1.74±1.34	0.1
Successful conservative treatment	64 (73.56)	12 (20.0)	0.01
Operative rate (surgery)	23 (26.43)	48 (80.0)	0.01
Hospital stay in non-operative patients (days)	3.1±1.8	2.6±1.3	0.17
Dye Reaching Colon (within 24 hours)	69 (79.31)	23 (26.4)	0.05
No Dye in Colon (within 24 hours)	18 (20.68)	8 (9.19)	0.01
Hospital stay (days)	6.5±2.1	5.4±2.3	0.37
In-hospital morbidity, n (%)	8 (13.3)	12 (20.0)	0.09
In-hospital mortality, n (%)	0	2 (3.3)	0.27

## References

[1] Tong JWV (2020). Acute Med Surg..

[2] Ghimire P (2023). J Nepal Med Assoc..

[3] Jang Y (2022). Ann Coloproctol..

[4] Klingbeil KD (2022). Surg Open Sci..

[5] Gu L (2021). Med Sci Monit..

[6] Almafreji I (2020). Cureus..

[7] Haule C (2013). J Clin Trials..

[8] Catena F (2016). World J Gastrointest Surg..

[9] Wesselink E (2018). Clin Nutr ESPEN..

[10] Bonnard A (2011). Pediatr Surg Inter..

[11] Choi HK (2002). Ann Surg..

[12] Biondo S (2003). Brit J Surg..

[13] Choi HK (2005). World J Gastroenterol..

[14] Di Saverio S (2008). World J Surg..

[15] Abbas SM (2007). Br J Surg..

[16] Choi HK (2002). Ann Surg..

[17] Cox MR (1993). Aust NZ J Surg..

[18] Matter I (1997). Eur J Surg..

[19] Sosa J (1993). Am Surg..

[20] Fuzun M (1991). Br J Surg..

[21] Ellis H. (1997). Eur J Surg..

